# Parallel universes of Black Six biology

**DOI:** 10.1186/1745-6150-9-18

**Published:** 2014-07-19

**Authors:** Alexander Kraev

**Affiliations:** 1 Charles H. Best Institute, Banting and Best Department of Medical Research, University of Toronto, Toronto, Ontario M5G 1L6, Canada

**Keywords:** Mouse, Inbred strain, C57BL/6J, Synthetic lethality, Gene interaction, Essential gene, Nicotinamide nucleotide transhydrogenase, Mitochondria, Glucocorticoid hormone, Adrenal gland

## Abstract

Creation of lethal and synthetic lethal mutations in an experimental organism is a cornerstone of genetic dissection of gene function, and is related to the concept of an essential gene. Common inbred mouse strains carry background mutations, which can act as genetic modifiers, interfering with the assignment of gene essentiality. The inbred strain C57BL/6J, commonly known as “Black Six”, stands out, as it carries a spontaneous homozygous deletion in the nicotinamide nucleotide transhydrogenase (*Nnt*) gene [GenBank: AH009385.2], resulting in impairment of steroidogenic mitochondria of the adrenal gland, and a multitude of indirect modifier effects, coming from alteration of glucocorticoid-regulated processes. Over time, the popular strain has been used, by means of gene targeting technology, to assign “essential” and “redundant” qualifiers to numerous genes, thus creating an internally consistent “parallel universe” of knowledge. It is unrealistic to suggest phasing-out of this strain, given the scope of shared resources built around it, however, continuing on the road of “strain-unawareness” will result in profound waste of effort, particularly where translational research is concerned. The review analyzes the historical roots of this phenomenon and proposes that building of “parallel universes” should be urgently made visible to a critical reader by obligatory use of unambiguous and persistent tags in publications and databases, such as hypertext links, pointing to a vendor’s strain description web page, or to a digital object identifier (d.o.i.) of the original publication, so that any research done exclusively in C57BL/6J, could be easily identified.

**Reviewers:**

This article was reviewed by Dr. Neil Smalheiser and Dr. Miguel Andrade-Navarro.

## Introduction

The concept of an essential gene, whose targeted inactivation results in lethality of a genetically modified organism, is a cornerstone of genetic dissection of gene function. Also important is the derivative concept of synthetic lethality, wherein inactivation of either of any two genes is not lethal, but simultaneous inactivation is lethal. Although synthetic lethality was reportedly discovered in fruit fly *Drosophila* as early as in 1922 (historical account [1]), Theodosius Dobzhansky first made extensive use of it and, indeed, coined the term. He wrote [2] in his 1945 paper: “Chromosomes A and B evidently contain genes, or groups of genes, which taken separately are not lethal to homozygotes raised at 16.5 degrees, but which become lethal when combined by crossing over. In view of their known origin by crossing over, chromosomes Nos. 18, 41, and 63 may be said to carry “synthetic lethals.” Ultimately, an exhaustive gene network can be constructed, if all synthetic lethal phenotypes are analyzed [3], aiding in post-genomic methods of drug discovery [4]. Considerable progress in analyzing “lethal combinations” [5] has been achieved in bacteria and yeast [6,7], and in the nematode *C.elegans* (recent review [8]) but the corresponding analysis in mammals lags behind.

A major portion of our current knowledge about gene function in mammals is derived from the phenotypes of mouse strains with targeted or random gene inactivation [9]. The mouse research community enjoys the availability of an extensive palette of standardized inbred strains, as well as strains with targeted inactivation/mutation of specific genes, and many of these are commercially available from established sources (The Jackson Laboratory, Charles River, Harlan, Taconic Farms, to name a few). An ambitious project aimed at inactivation of all predicted mouse genes is in progress [10]. Even though mouse models of disease are not limited to targeted gene mutation or inactivation, the results of studies of the strains produced by gene targeting technology have made an undisputable impact on biology and medicine.

In recent times, it has been found that the most common inbred mouse strains carry background mutations [11-18] that conceivably interact with phenotypes created by targeted gene disruption, thus possibly interfering with the assignment of “essential” and “redundant” gene qualifiers. Such mutant alleles are among “modifier genes”, the case of cystic fibrosis CFTR gene being the classical example (recent review [19]), some of which are capable of altering the phenotype of a large number of other mutations. Beside the large number of known modifiers with a limited effect (recent review [20]), a few point mutations with implied systemic effect have been uncovered in specific groups of strains by modest, gene-focused approach [11-18]. In addition, a much larger number of differences between the genomes of laboratory mouse strains, mostly with unclear consequences, have been discovered since the application of next generation sequencing [21-24]. Genome changes that occurred during the rodent lineage specification, such as large segmental duplications ([23,24] and references therein), can now be clearly distinguished from relatively late changes in the mouse genome that have occurred since domestication, presumably as the result of adaptation to a life in captivity and in production facility [25,26].

The inbred strain C57BL/6J, commonly known, along with its other substrains, as “Black Six” stands out, as it carries a homozygous spontaneous deletion in the *Nnt* gene [12,27]. The product of this gene [GenBank: AH009385.2], by virtue of its expression profile [28] directly affects the function of steroidogenic mitochondria [29] and through the function of the hypothalamic-pituitary-adrenal axis, adrenal response to stress (for a recent succinct review, see [30]). At the same time, the strain has been used by means of gene targeting technology to assign “essential” and “redundant” qualifiers to numerous genes. The purpose of this review is not to raise another concern about the importance of exact mouse nomenclature, but to expose the particular danger of the *Nnt* mutation, which, due to its underappreciated wide systemic effect, has aided in creation of an internally consistent “parallel universe” of knowledge, growing at an alarming rate. The review analyzes the historical roots of this phenomenon and proposes that building of “parallel universes” should be urgently made visible to a critical reader by obligatory use of unambiguous animal strain identifiers in publications and databases, such as hypertext links pointing to a vendor’s strain description web page (including stock number), or to a digital object identifier (d.o.i.) of the original publication.

## Review

### The power of Black Six

In March 1998, a panel of international scientists met to make recommendations to the National Institutes of Health (USA) regarding priorities for generating mouse genomics and genetics resources. A consequence of these recommendations was the general approval of a strategy to sequence the mouse genome. The action plan for mouse genomics was published in Nature Genetics [31].

Although there was considerable debate in the research community about which mouse strain should ultimately be used to derive the index mouse genome sequence, the Coordinating Group unanimously decided on October 5, 1998 that the best choice was the C57BL/6J strain, commercially available from The Jackson Laboratory. The reason for selecting C57BL/6J out of a palette of other popular strains was “confidence of strain derivation, widespread use among the research community and favorable breeding characteristics” [31]. Widespread use of this strain was, in large part, determined by established targeted mutation technology [32-34], wherein the strain was routinely used as a host for genetically transformed cells, derived from a 129-family strain. In 2002, C57BL/6J became the first mouse strain with a sequenced genome, albeit with many gaps, and also the first mammalian model organism with a reference genome. This was carried out in contrast to the way in which the human genome sequence was derived [35,36]; the human genome was a consensus of several genetically distant individuals. In 2009 a new, considerably refined, C57BL/6J genome sequence was published [23].

These events had a snowball effect and reinforced the use of the C57BL/6J strain in the mouse research community. Publications show that many laboratories started to move their mutant genes of interest to the C57BL/6J background, even when they may originally have been obtained in a different strain. In 2013 this strain was introduced into space research [37,38].

As the great majority of laboratories were not equipped to make genetically modified strains on their own, but rather collaborated to create such a strain, borrowed or purchased it, a logistics pyramid emerged spontaneously to ensure the widespread use of the C57BL/6J strain throughout the research community. For a substantial portion of it there really was no choice in selecting the C57BL/6J strain due to the existence of the logistics pyramid. Moreover, many in the research community probably did not care about the reference strain identity and its genetic background, an impression that can be gleaned from the frequent absence of strain identity or consistent strain misspellings in publications.

There was, however, a downside to the widespread use of C57BL/6J for genetic studies: the phenotype of a targeted mutation on the C57BL/6J strain sometimes displayed peculiar characteristics, in particular, where comparable data on the properties of the same mutation on a background of another strain were available. One of the early examples of this kind is the knockout phenotype of the glucose-6-phosphate dehydrogenase gene [39], which is lethal on the C57BL/6J background, but not so on the hybrid C57/129 background. In humans, this gene is one of the most frequently mutated, particularly in African populations, as the reduced activity of the protein product confers protection from malaria [40]. From this comparison it could be concluded that this gene is “essential” only in C57BL/6J, though “redundant” in other strains, or in humans. The authors did not suspect that they were observing a case of *synthetic lethality*. Such discrepancies, which accumulated only slowly, were usually dismissed, so the fecund and sociable C57BL/6J did not stand out in any particularly unfavorable way at the beginning of the 21^st^ century.

#### Mutatis mutandis?

Perhaps the first mutation with an implied pleiotropic effect found in inbred mouse strains was the inactivation of the gene, encoding aralkylamine N-acetyltransferase, *aanat*[11]. The inactivation results in the absence of synthesis of melatonin, a pineal gland hormone [41,42]. While it affected C57BL/6J and a number of other strains, it was not immediately apparent how this mutation might stand in the way of progress, as chronobiology was still in its infancy. In contrast, the discovery in 2001 of a large genomic deletion in a C57BL/6 lineage, offered commercially by Harlan, invoked a short, but emotional letter on the importance of exact strain nomenclature [43]. The importance of adherence to exact strain nomenclature, as well as the issue of genetic background was, indeed, raised more than once [44-48] but was largely ignored. In 2005, Toye et al. [12] found, using a classical genetic mapping approach, that glucose intolerance in C57BL/6J is largely due to a naturally occurring “knock-out” of the *Nnt* gene, encoding nicotinamide nucleotide transhydrogenase, a nuclear-encoded mitochondrial proton pump [49]. This publication invoked a special commentary on the web site of the strain vendor [50], but did little to alter practices in the research community. More recent publications [51,52] showed that a twin strain C57BL/6N did not carry the *Nnt* mutation, although it was a descendant of the same original strain from which C57BL/6J was derived (historical account [53]). However, gene targeted strains had been created on both of these backgrounds, often without explicit knowledge of the differences between them [54]. The Navarro et al. study checked 79 randomly picked gene-targeted strains deposited to The Jackson Laboratory and found that 19 of them were homozygous *Nnt*^*+/+*^*,* 7 heterozygous *Nnt*^*+/-*^ and the rest homozygous *Nnt*^*-/-*^*.* Sadly, even these discoveries did not appear to sound an alarm throughout the worldwide mouse community, and neither did similar publications showing that other deleterious mutations were present in common mouse strains (mentioned in the *Introduction*). The pattern is recognizable in the public databases and resources, i.e. the comprehensive mouse embryonic stem cell mutant resource by the International Knockout Mouse Consortium uses the *Nnt*^*+/+*^*,* strain C57BL/6N [10], while C57BL/6J is used by the extensive brain-specific gene expression atlas of the Allen Institute for Brain Science [55] and the three-dimensional brain MRI atlas [56].

However, behind the scenes, the mouse community became divided into two broad groups. One group continued to ignore the problem of background mutations, often reporting inexact or obscure citation of the strain name, or burying its identity in a chain of references to the previous papers. The second group of researchers, recognizing the problem and the potential in comparing the phenotype on more than one background has started to move their targeted mutations to another background, such as the similar, but *Nnt*^*+/+*^ strain C57BL/6N, or to a distant background, with an attempt to map the genetic modifiers of the original phenotype.

Publications since 2012 of multiple mouse strain sequences derived by next generation sequencing technology have revealed a number of point mutations and insertions/deletions in many, if not all, of the popular strains, the scope of which does not correlate with the apparent “normality” of the mice [21]. Such discoveries question the importance of studies of the point mutation of just one peculiar gene. However, by now substantial data has accumulated that the peculiar properties of certain targeted mutations on the C57BL/6J background are not seen when the mutation is expressed on the background of other popular strains such as FVB/N or 129/Sv, or mixed (hybrid) mouse backgrounds, or even in the patient, whose disease was modeled in the mouse. Such examples are too numerous to be comprehensively included here, without a bias toward a specific field or research group.

Importantly, strain specificity of gene-targeted mutations has been seen in such processes as glucocorticoid synthesis [57] and transport [58-61], as well as programmed cell death [52,62], all known to involve the endoplasmic reticulum and/or mitochondria. However, while some authors began to suspect the strain background when mouse model results did not match the results on human patients with the analogous mutation [63], numerous other authors did not question the results obtained only on C57BL/6J [64-72], and some even defended the superiority of this strain and considered the results obtained on other strains as problematic [73]^a^.

#### *Nnt* deletion in C57BL/6J has more than one systemic endophenotype

Independent research in patients has shown that NNT is the fourth gene associated with familial glucocorticoid deficiency (OMIM #614736) in humans [29]. This discovery also revealed that *de facto,* an unplanned international experiment in the mouse has been going on for 3 decades, in which many targeted mutations on C57BL/6J background have unintentionally been combined pairwise with a pleiotropic mutation, often resulting in *synthetic* lethality.

A recent study in four European magnetic resonance imaging centers found that C57BL/6J also displays sporadic porto-systemic shunts (a liver dysmorphology), which were originally discovered as unexplained spikes of neurotransmitter glutamate in its brain [74]. This condition is also known in humans, and patients with it display multiple neuro-behavioral problems [75]. This discovery puts the C57BL/6J strain in the problematic category with respect to its utility for behavioral studies.

### Parallel universes of Black Six biology

The background *Nnt* mutation in C57BL/6J stands out in comparison to other known strain-specific mutations because it affects the so-called hypothalamic-pituitary-adrenal axis, a complex and incompletely understood system that regulates multiple processes at the whole organism level (recent review [76]). Consequently, its effects may be hard to detect in the reference strain itself in the absence of “stress” [77,78]. The *Nnt* mutation ablates a protein expressed in mitochondria of many tissues [28] and thus is likely to interact with a large number of other targeted mutations. Parenthetically, mutations, introduced by gene targeting, might also be viewed as a form of stress. The absence of the *Nnt* protein in steroidogenic mitochondria in the adrenal gland results in a multifaceted phenotype, presumably due to altered corticosteroid signaling, which is, perhaps, still incompletely characterized. Regardless of the mechanism, it is much more important that, if a result of gene targeting appeared to be embryonic lethal in C57BL/6J, the respective targeted gene qualified as “essential”. On the other hand, if a gene ablation did not result in lethality, the gene was labeled as “non-essential” and the respective protein function was considered to be “redundant”. The scale of this massive misclassification of genes as “essential” or “redundant” has progressed to the point of creating a consistent C57BL/6J-specific “parallel universe” of knowledge about gene function, a “Black Six biology”.

Evidently, growth of “parallel universes” could go undetected for a long time, as a similar study is not often done in parallel in different experimental organisms, and even if discrepancy arises, it can usually be attributed to “species difference” or, not quite as often, to “genetic background effects” or to “undocumented confounders”. The consequences are more serious in the cases where mouse research produces robust leads for the so-called “translational research”. The ignorance of the possibility of the genetic background effects could result in two scenarios. First, it can make a useless drug go further into clinical studies, only to be found dangerous in the toxicity tests. Second, it could preclude a promising drug from crossing the great translational divide, also known as the ”Valley of Death” [79-83], separating the pre-clinical and clinical studies. Both scenarios will likely result in a profound waste of effort, rather than progress to a true medical catastrophe.

By extension, this conclusion also applies to other popular strains having other deleterious mutations, except that none of the other strains comes close to the situation with the *Nnt* mutation in C57BL/6J, if one considers the pleiotropic nature of the lesion and the historical strain popularity combined.

Recently, a comparative genomic and phenotypic analysis of the twin strains, C57BL/6J and C57BL/6N, has been published by a consortium of 18 European laboratories [46,53]. While this huge study is much more than a reiteration of concern about strain nomenclature, it carefully downplays the scale of the *informational* consequences of the systemic phenotype(s) of the *Nnt* mutation and only tentatively suggests that the effect of this mutation alone could supersede the more modest effects of the other genomic differences found between these twin strains.

### What should be done?

This review is not an advocacy of a worldwide ban on C57BL/6J, nor is it a call for massive retraction of publications using this strain. However, certain pivotal experiments done exclusively on C57BL/6J in the past should be *validated* on a progeny of an F1 cross with a distant strain, to verify that these discoveries do not belong to “parallel universes” of Black Six biology. By doing so, one might hope to eventually reap some of the benefits of the unplanned large scale international experiment spanning over three decades, wherein numerous gene targeted mutations have been combined pairwise with the pleiotropic effect of the *Nnt* mutation.

The building of the “parallel universes” should and can be put under control. The important and urgent step would be to implement a modern mode of tagging of the available information. Instead of current lax approach, I would suggest the use of unambiguous strain identifier, such as a hypertext tag pointing to the vendor’s description web page (if the strain is commercial), or to the “digital object identifier” (d.o.i.) of the original publication, if the strain was obtained by other means. Here is how the strain C57BL/6J could be identified by the hypertext link: C57BL/6J [http://jaxmice.jax.org/strain/000664.html]. It would be even more appropriate, if a vendor-independent Reference Laboratory Strain Database could be created, to which the hypertext links could be directed. For now, it is within the power of such public databases as NCBI’s PubMed to introduce an obligatory strain identity tag on every on-line abstract. Ideally, journal editors should be the ones to elevate the correct strain information to the same level of importance as currently applied to chemical formulae and nucleotide/protein sequences*.* Currently, despite the existence of the ARRIVE guidelines for animal research [84] that emphasize the importance of correct animal identity, only a few journals have actually adopted them.

## Conclusions

If Hans Selye [85] were alive today, he would have certainly been deeply concerned by the fact that a mouse strain with a remodeled stress response and an impaired hypothalamic-pituitary-adrenal axis is used in the 21^st^ century as a reference in hundreds of laboratories worldwide. The altered hypothalamic-pituitary-adrenal axis response affects not only the assignment of the “essential” and “redundant” gene qualifiers, on which this review was deliberately focused, but also non-hereditary mouse models, such as those with chemically induced conditions.

I can tentatively attribute the pervasive “strain unawareness” to at least two dissimilar reasons. The first reason could be called “shadow of Claude Bernard” [86], a legacy of physiological paradigms of the 20^th^ century. Indeed, genetic identity of experimental organism (or lack thereof) did not concern physiology for the most part of the 20^th^ century [87], wherein many fundamental experiments were done on mongrel dogs. Today, mouse models are still created within this school of thought. The second reason is that the unique system of shared resources and associated institutions in mouse research, created throughout the 20^th^ century, acts as a powerful incentive for fallback on a single strain, C57BL/6J. Consequently, suggesting a gradual phasing out of this strain is unrealistic. This situation is aggravated by the existence of “twin” substrains, C57BL/6J and C57BL/6N, having different systemic mutations [12,18], and by the fact that even though a study of the *Nnt* genotype of the commercially available C57BL/6 substrains has been published [88], the vendors have yet to follow the example of The Jackson Laboratory and Charles River and include the *Nnt* genotype in their strain descriptions.

A recent proteomics study [89], comparing the response of two common mouse strains, C57BL/6J and 129/Sv, to high-fat diet, is not only a pertinent example of just how different the same physiological reaction could be in two distantly related mouse strains, but also an appropriate test question: which of the two strains should one trust in anti-obesity drug testing? If one still thinks “C57BL/6J”, this review has not reached its goal. In this author’s opinion, the correct answer is: the F1 hybrid of the two, since it precludes the recessive background mutations of either strain from affecting the outcome.

As for basic research, it is important to understand that no number of new experiments targeting individual genes can make the “parallel universes” of knowledge quietly vanish, but continuing on the road of “strain unawareness” slowly *annihilates* a substantial portion of the knowledge that has been accumulated, creating controversies, going to the point where trust in mouse as an experimental organism would falter.

Finally, perhaps, the story of C57BL/6 “twins” could be a hint that maintenance of an inbred strain for hundreds of generations is not the best way of assuring consistency. Today, armed with the refined genome sequence and contemporary genome analysis tools, geneticists could consider repeated rederivation “from ground up” of an entirely new, post-genomic, reference strain.

## Endnote

^a^While this selection of references constitutes a possible bias toward specific fields and research groups, the intention was to provide a sufficient breadth of examples with potential clinical implications, without excessive referencing. This selection is by no means complete or driven by personal reasons.

## Reviewers' comments

### Reviewer 1 report

Dr. Neil R. Smalheiser, University of Illinois at Chicago, United States of America

This is a fascinating and scholarly account of “Black Six biology” with lessons for anyone using inbred mice in biology. I think it will be widely read and discussed. However, I do strongly suggest making a number of changes:

First, the tone of the paper begins as a scholarly discussion, then changes abruptly in the Conclusions section, becoming negative, polemical and defensive. This posture is not warranted and will not move readers to the desired actions. I would remove loaded words such as “shocked”, “deranged”, “obviously defective”, and “folly”. Moreover, I disagree that there has been any suppression of dissident views on the subject of inbred strains. (The long list of references cited in this paper, including Wotjak’s paper in the prominent Trends in Genetics, is evidence to the contrary.) I agree that research results often take a long time to result in changes in behavior: think of how difficult it was to get physicians to wash their hands between patients, or to convince scientists to take seriously the threat of contamination of their cell lines by HeLa. Often information is not enough to change behavior, and perhaps circulating this paper will help, but claiming suppression will definitely NOT help.

Author's response: *I am very grateful to the reviewer for his critical analysis of the manuscript and constructive comments. Indeed, correct word choice is very important for the message to reach the audience without alienating anybody. I have changed the words “shocked”, “deranged”, “obviously defective” to “deeply concerned”, “remodeled” and “impaired” respectively. The term “black mouse folly” has been replaced with “strain unawareness”. I removed the reference to suppression of dissident views, which was clearly inadequate*.

Minor changes: a) The last sentence of the abstract is hard to understand, and should be rewritten in plainer English. b) Is ANY single inbred strain of mouse likely to be perfect for carrying out knock out experiments? If not, then you should clarify better that it is not 6J that is the problem, but the lack of robustness built into the experiments (i.e. perhaps lethality should be an outcome averaged over a number of diverse strains?). Statements such as “interfering with the assignment of “essential” and “redundant” gene labels” (p. 3) or “massive misclassification of proteins as “essential” or “redundant” (p. 9) should be changed, since any assignment is relative to some choice of strain and some background genome. c) Please define synthetic lethality (p. 6). d) The term “judicial” (p. 11) is obscure and should be replaced with something easier to understand.

Author's response: *Although the suggestions are listed by the reviewer as minor, they prompted me to rewrite portions of the Introduction and move a portion from the Conclusions into a new section, particularly that similar concerns were raised by the other reviewer. Specifically,*

a) the last sentence in the revised Abstract now reads: “…proposes that building of “parallel universes” should be urgently made visible to a critical reader by obligatory use of unambiguous and persistent tags in publications and databases, such as hypertext links, pointing to a vendor’s strain description web page, or to a digital object identifier (doi) of the original publication, so that any research done exclusively in C57BL/6J, could be easily identified.”

b) The short answer is that no single current strain is perfect for all conceivable knockout experiments, however, giving practical advice on strain selection was not the purpose of my review, instead, the focus is on the legacy of “strain unawareness”. I stand by the idea that the legacy of strain unawareness is massive functional misclassification of genes. Besides, since controls for background-specific phenomena (including lethality), such as outcross to a genetically distant strain are often not done, indeed there is also a common lack of robustness in mouse experiments, presumably dictated by their cost. However, while technically gene essentiality assignment always refers to a specific (genetic) background, as long as it is currently accepted that core physiology of all mammals is the same, consequently certain genes should be essential in mammals regardless of background.

*c) expanded historical background and a definition of synthetic lethality is now provided in the Introduction*.

d) word “judicial” was replaced by “modern mode”.

### Reviewer 1 report on the revised version

The paper is improved and reads well. I would suggest making a few minor changes of wording: a) In the section What Should be Done?, I would remove the phrase "as some of my previous reviewers have suggested". b) In the section Conclusions, "Had Hans Selye been alive" is improper grammar [certainly he had been alive]. Maybe say something like, If Hans Selye were alive today,.... c) In the third paragraph of Conclusions, I would rephrase or remove the first sentence (that includes "medicine is not in immediate danger of the invasion of newdrugs,…).

Author's response: *I accept all of the reviewer’s suggestions. The text has been changed to accommodate them.*

## Reviewer 2 report

Dr. Miguel A. Andrade-Navarro, Max Delbrück Center for Molecular Medicine, Germany

The manuscript from Kraev points to a serious issue affecting 30 years of research in mouse genetics: the widespread use of the inbred strain C57BL/6J (or Black Six) with known and often ignored genetic and physiological defects, and the failure of many reports to indicate whether they use this or other mouse strains. As a result, the relevance of many published experimental results remains questionable until these experiments are repeated in mouse strains of “more robust” genetics. The author proposes that mouse strains should be clearly annotated in publications, and that the use of Black Six for genetics research should be carefully considered. While, as he admits, he is not the first to state these claims, this manuscript collects the bibliography relevant to the problem and insists on the annotation approach.

Major comments

The author needs to be more clear about whether he proposes that mouse strains must be tagged, or some strains not be used, or what. I think he suggests both and then this should be stated in the abstract.

Author's response: *I am grateful to the reviewer for in-depth analysis of the manuscript and many constructive suggestions. I do suggest that mouse strains should be more clearly tagged in publications, but I do not suggest any kind of prohibition on certain strains. Rather, the authors should be aware of the background mutations and their possible effect on their studies. Ideally, more than one strain should be used to evaluate a newly discovered effect of a targeted gene modification (knock-in or knock-out), particularly the one that results in lethality.*

The technical details of how to annotate mouse strains in future and past manuscripts are loosely defined as “hypertext tagging”. In the absence of a deeper discussion on how the annotations will be achieved, I suggest to be simpler and I would change the sentence in page 2: “by obligatory hypertext tagging of proper use, as well of as misuse and omission, of exact animal strain names in publications and databases.” to “by obligatory tagging of exact animal strain names in publications and databases.”

Author's response: *I agree with this comment. In the revised text, I give specific suggestions. Please also see below the response to “Page 4” comment on the proposed format of the strain tag.*

Also in page 11, I would simplify “This should start with biomedical literature, so that experiments done exclusively on C57BL/6J are clearly tagged with hypertext links and become identifiable for subsequent machine analysis.” to “This should start with biomedical literature, so that experiments done exclusively on C57BL/6J are clearly tagged.”

Author's response: *I agree with this suggestion, the Abstract and the body of the text have been changed to accommodate it.*

Then, the author might need to be more specific about whom is he addressing: are journal editors supposed to enforce the proper description of mouse strains in new papers? Should researchers stop using certain strains for particular topics of research? Should database centers like the NCBI and the EBI back-annotate existing bibliographic records (e.g. MEDLINE) to include missing strain annotation? Should authors of published literature contribute to annotate their database records?

Author's response*: Ideally, journal editors should be the ones to elevate obligatory strain designation to the same importance level as chemical formulae and nucleotide/protein sequences. Strain identity is one of the central issues in the ARRIVE guidelines, published in 2010, which are, admittedly, voluntary, and are currently supported by only a few journals. In this review, I did not plan to elaborate on which strains are bad choices for specific fields of research, it is an attractive topic for more focused future publications. It would certainly be desirable that database centers back-annotate existing bibliographic records, but this needs a specialized discussion, which hopefully follows after the publication of this review. The back-annotation would obviously be incomplete without active participation of the authors of the original research.*

Regarding database records, there are already MeSH terms for mouse strains that are used to annotate records in MEDLINE. See for example term: Mice, Inbred C57BL [B01.050.150.900.649.865.635.505.500.400.420]. There are many others that the author could examine at: http://www.nlm.nih.gov/cgi/mesh/2014/MB_cgi?mode=dcms&term=Mice,+Inbred+Strains&field=entry#TreeB01.050.050.157.520

If the author thinks that these terms are incomplete then he could say so and suggest how these terms should be completed.

Author's response*: I have expanded a portion of the Conclusions into a separate section to discuss this point. Indeed, relevant MESH terms are useful, but they are in need of update. The reference, suggested by the reviewer, contains MESH terms for only a few most common mouse strains, but does not allow distinction between the strains of the C57BL family, two of which are the topic of this review.*

Page 4: “The purpose of this review is not to raise another concern about the importance of exact mouse nomenclature, but to expose the particular danger of a single mutation, which has aided in creation of an internally consistent parallel universe of knowledge. I suggest a simple way to stop this trend.” May be it would help here to explain shortly what the suggestion is.

Author's response*: I created a new subsection to elaborate on this point. Essentially, it amounts to the use of unambiguous strain identifier, such as a hypertext tag pointing to the vendor’s description web page (if the strain is commercial), or to the “digital object identifier” (doi) of the original publication, if the strain was obtained by other means. Here is how the strain C57BL/6J would be identified by the hypertext link*: C57BL/6J [http://jaxmice.jax.org/strain/000664.html].

Page 9. While I am sympathetic with the point being made, I am not sure to agree with the dramatic example given in page 9: “if a new drug for human consumption were suggested as a result of experimentation in C57BL/6J alone [?]”. There are so many differences between mice and humans that I doubt the “abnormalities” of Black Six would specifically result in dangerous drugs. In general I would here just insist on the danger of extracting conclusions on booby-trapped genetic backgrounds.

Author's response*: The anticipation of dramatic consequences, possibly associated with this problem, hinges on how much one believes in the existence of the great translational divide, also known as the“Valley of Death”, which separates pre-clinical studies from the clinical ones. I expanded this portion into suggesting that the systemic defect, such as glucocorticoid deficiency, can both make a seemingly promising drug to go further into clinical studies and subsequently rejected, as well as, more importantly, make an actually promising drug to fail in animal studies, and thus prevent it from crossing the translational divide. Either outcome will likely result in a wasted effort, rather than invoke a medical catastrophe, so, indeed, the original drama was exaggerated.*

Minor comments

The first sentence in abstract and introduction are the same. This is a bit distracting. I would change either.

Author's response: *The phrases have been changed*.

Page 3: “Even though mouse models of disease are not limited to targeted gene mutation or inactivation, it has made an undisputable impact on biology and medicine.” This sentence is not clear. What is “it” referring to?

Author's response*: This phrase appeared as an error of article shortening. I am grateful to the reviewer for pointing to the ambiguous phrase, which has been corrected.*

A number of references seem not to be cited in the main manuscript and could be removed from the reference list. In the current version these are Ref 16 (page 3), Refs 30-31 (page 5), Ref 43 (page 6),

Author's response*: Orphan references were removed and the reference list is updated to reflect the revised text.*

## Competing interests

The author declares that he has no competing interests.
